# Analysis of the recovery phase after maximal exercise in children with repaired tetralogy of Fallot and the relationship with ventricular function

**DOI:** 10.1371/journal.pone.0244312

**Published:** 2020-12-18

**Authors:** Ilse Coomans, Sara De Kinder, Hannah Van Belleghem, Katya De Groote, Joseph Panzer, Hans De Wilde, Laura Muiño Mosquera, Katrien François, Thierry Bové, Thomas Martens, Daniël De Wolf, Jan Boone, Kristof Vandekerckhove

**Affiliations:** 1 Department of Pediatric Cardiology, Ghent University Hospital, Ghent, Belgium; 2 Faculty of Medicine, Ghent University, Ghent, Belgium; 3 Department of Cardiac Surgery, Ghent University Hospital, Ghent, Belgium; 4 Department of Movement and Sports Sciences, Ghent University, Ghent, Belgium; University of Minnesota, UNITED STATES

## Abstract

**Background:**

Few studies demonstrate delayed recovery after exercise in children and adults with heart disease. We assess the recovery patterns of gas exchange parameters and heart rate (HR) in children with repaired Tetralogy of Fallot (rToF) compared to healthy peers and investigate the correlation with ventricular function and QRS duration.

**Methods:**

45 children after rToF and 45 controls performed a maximal incremental cardiopulmonary exercise test. In the subsequent recovery period, patterns of VO_2_, VCO_2_ and HR were analysed. Half-life time (T_1/2_) of the exponential decay and drop per minute (Rec_min_) were compared between groups. In the rToF group, correlations were examined between the recovery parameters and QRS-duration and ventricular function, described by fractional shortening (FS) and tricuspid annular plane systolic excursion (TAPSE) measured at baseline prior to exercise.

**Results:**

Recovery of VO_2_ and VCO_2_ was delayed in rToF patients, half-life time values were higher compared to controls (T_1/2_VO_2_ 52.51 ±11.29 s vs. 44.31 ± 10.47 s; p = 0.001 and T_1/2_VCO_2_ 68.28 ± 13.84 s vs. 59.41 ± 12.06 s; p = 0.002) and percentage drop from maximal value was slower at each minute of recovery (p<0.05). Correlations were found with FS (T_1/2_VO_2_: r = -0.517; p<0.001; Rec_1min_VO_2_: r = -0.636, p<0.001; Rec_1min_VCO_2_: r = -0.373, p = 0.012) and TAPSE (T_1/2_VO_2_: r = -0.505; p<0.001; Rec_1min_VO_2_: r = -0.566, p<0.001; T_1/2_VCO_2_: r = -0.466; p = 0.001; Rec_1min_VCO_2_: r = -0.507, p<0.001), not with QRS-duration. No difference was found in HR recovery between patients and controls.

**Conclusions:**

Children after rToF show a delayed gas exchange recovery after exercise. This delay correlates to ventricular function, demonstrating its importance in recovery after physical activity.

## Introduction

Tetralogy of Fallot (ToF) is one of the most frequent congenital cardiac defects accounting for 3–10% of newborns with congenital cardiopathy. Surgical repair is usually performed within one year of age with excellent long-term outcome [1, 2]. Nevertheless, exercise testing shows that maximal exercise performance in patients after repair of ToF (rToF) remains impaired with VO_2_ peak values usually about 20% lower than healthy peers [3]. Cardiopulmonary exercise testing (CPET) is a well-established tool in the assessment of the cardiovascular function and has proven to have prognostic value, besides QRS duration, in the decision for reintervention and the prevention of premature sudden death [4].

The recovery after maximal exercise in children has been poorly investigated in literature. It is known that healthy children recover faster than adults [5, 6] but data about the recovery after CPET at childhood age in patients with congenital heart defects (CHD) is limited. It has already been demonstrated that the recovery kinetics of gas exchange parameters and heart rate are prolonged in patients with different types of CHD [7, 8]. In adolescents and adults, impaired right-sided haemodynamic and central autonomous nervous activity lead to a delay in cardiovascular recovery and it has been suggested that these observations could be important to guide reintervention [9, 10]. However, most studies focus on adult populations or examine heterogeneous groups with mixed cardiopathies. To our knowledge no study has been published focusing solely on the recovery kinetics in young children after repair of ToF in comparison to healthy children.

The main purpose of our study was to evaluate the recovery after maximal exercise in children between 6–18 years of age with repaired ToF. We compared the recovery patterns after maximal exercise between rToF-patients and healthy matched controls. We hypothesize that children with rToF have an impaired recovery capacity and thus recover more slowly. In addition, we investigated whether parameters of cardiac function or QRS-duration are related to the recovery pattern in this specific patient group. Ultimately, better insight into the recovery phase and its determining factors might provide additional information useful in guiding further treatment.

## Materials and methods

### Study population

In this retrospective study, patients between 6 and 18 years with repaired Tetralogy of Fallot (rToF) who were treated at the department of Paediatric Cardiology at the Ghent University Hospital were included. They performed a cardiopulmonary exercise test as part of their routine follow-up between Jan 2013 and May 2018. Tests with a recovery period of less than 4 minutes were excluded.

Cardiac function was evaluated by echocardiography data obtained within the same month of the exercise test. Left ventricular function was assessed by fractional shortening (FS), right ventricular function by tricuspid annular plane systolic excursion (TAPSE) and calculated TAPSE z-score [11] (zTAPSE). The right ventricle was considered dilated when the RV/LV ratio on apical 4 chamber view was larger than 1 (RVdil). QRS-duration was obtained from the resting ECG at the moment of the exercise test. Data on pulmonary valve replacement (PVR), PV stenosis and insufficiency were collected from the patient medical file.

The control group consisted of children who performed an exercise test in that same period. They were referred for minor complaints during exercise, such as chest pain, palpitations or breathing difficulties, but were cleared negative. The controls were selected to match one-on-one with the patient population with regards to each of the following parameters: gender, age, weight and length.

Written informed consent was obtained from one of the parents or the legal guardian. For children older than 12 years, assent from the children was also obtained. This study was performed in conformity with the guidelines of the Declaration of Helsinki and was approved by the ethical committee of the Ghent University Hospital (ref. B670201940069).

### Cardiopulmonary exercise test

An incremental exercise test was performed on an electromagnetically braked cycle ergometer (Ergoline Ergoselect 100K, Bitz, Germany). Following a 3-min warm-up at unloaded cycling, a ramp protocol was applied to increase the work rate in a linear and continuous way (i.e. ramp exercise). The ramp slope (i.e., the increase in work rate per minute) was individualized and determined by dividing the individual body weight by 4 and rounding off to the closest natural number (0 Watt+(body weight/4) Watt.min^-1^). Participants were asked to maintain a pedal rate of 60 rounds per minute (rpm) and the test was terminated when they reached their self-determined point of full exhaustion or were unable to maintain the required pedal rate despite strong verbal encouragement.

Measurements continued during a consecutive 6-minute recovery phase. In the first 2 minutes patients were asked to continue pedaling at a very low frequency and a workload equivalent to the one used at 1 minute of exercise to prevent fainting and accelerate lactate removal. In the following 4 minutes patients remained seated on the ergometer without pedaling.

Standard ventilatory and respiratory gas exchange parameters were obtained by dynamic breath-by-breath measurements (Oxycon Pro ergospirometry system, Jaeger, Hochenhausen, Germany). Before each test, the device was calibrated according to the manufacturer's instructions. Expired gas samples were analyzed for oxygen uptake (VO_2_, mL/min) and carbon dioxide production (VCO_2_, mL/min) and measurements were averaged per 5 seconds.

Twelve-lead ECG and heart rate (HR) were recorded continuously during the test, whereas blood pressure was recorded every 3 minutes during the exercise phase and every 2 minutes during the recovery phase with an integrated blood pressure monitor (SunTech Tango, Morrisville, NC) that uses 3D K-Sound Analysis.

### Data analysis

#### Exercise parameters

Peak values were defined as the average value over the last 10 seconds of the exercise phase. Since a levelling-off or a plateau in VO_2_ is often not reached in children [12], the term VO_2_peak will be used as a substitute for VO_2_max. The peak work load (Load) was determined as the work load attained at the termination of the exercise phase. The VO_2_peak and Load were expressed relative to the predicted values (PredVO_2_, PredLoad), obtained from the equations proposed by Wasserman, based on age and anthropometric values [13]. Respiratory exchange ratio (RER) was calculated using conventional equations (VO_2_/VCO_2_). Ventilatory efficiency was assessed by calculating the VE/VCO_2_ slope.

#### Recovery kinetics

Two different approaches were used to analyze the recovery phase kinetics of VO_2_, VCO_2_ and HR [5, 8, 14]. First, the relative change of VO2 and VCO2 was calculated after each minute of recovery as percentage drop from peak value. For example: the percentage change of VO_2_ after 2 minutes recovery (%Rec2-VO_2_) = 100%*(VO_2_ at 2min recovery—VO_2_peak)/VO_2_peak.

To characterize the recovery phase of HR, the HR decline from HRpeak was calculated in absolute values, for example HR drop after 2 minutes recovery (Rec2-HR) = (HR at 2min recovery -HRpeak).

**Fig 1:**
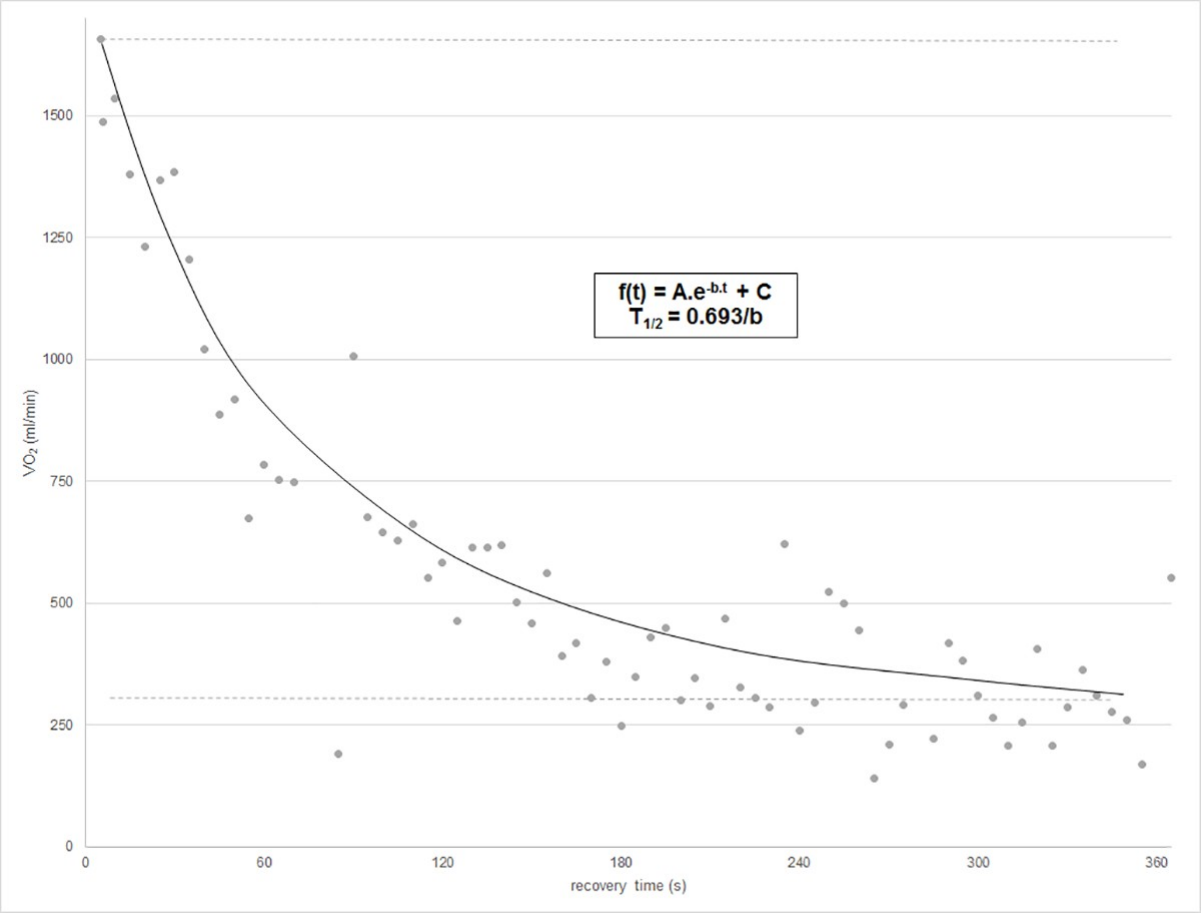
Example of VO_2_ response after maximal exercise. Data is fitted to a mono-exponential curve, as shown, and half-life time is calculated.

Secondly, the recovery kinetics of VO2, VCO2 and HR were considered as an exponential decay [5, 8, 14]. In Matlab (MathWorks, Natick, MA, USA) the dataset of the entire recovery phase was fitted to a mono-exponential equation

$$Y\left(t\right)=A.e^{‐b.t}+C$$

where Y(t) represents the parameter at the giving recovery time t (expressed in seconds), C is the baseline value, A+C is the peak value at the start of the recovery (t = 0s). The rate constant b quantifies the exponential decay. From b the more intuitive half-life time T_1/2_, i.e. the time needed to drop from peak value to half of the difference between peak and baseline value, can be calculated: T_1/2_ = 0.693/b. (Fig 1)

### Statistical analysis

SPSS v26.0 (IBM Corp., Armonk, NY, United States) was used to perform the statistical analyses. Results, unless stated otherwise, are expressed as mean ± SD. The normality Kolmogorov-Smirnov test with visual support of histograms was performed to determine whether continuous variables were normally distributed. If so, the differences between groups were evaluated by unpaired Student’s t-test. Otherwise, data were compared using the Mann-Whitney U test. The relation between variables was assessed by determination of the Pearson or Spearman coefficient.

To account for the repeated measures over time, a linear mixed model was fitted for VO2, VCO2 and HR using the restricted maximum likelihood approach with unstructured covariance pattern and with group (rTof vs control), time (minute 1 to 6) and their two-way interaction in the fixed part of the model. In case of a significant interaction term, post-hoc analysis was performed applying Bonferonni correction.

Statistical significance was set at P < 0.05.

## Results

Patient demographics are shown in Table 1. Ninety children performed a maximal CPET, 45 patients with rToF that met the proposed criteria could be selected and 45 controls were matched one-on-one accordingly. This resulted in two groups who were similar in gender distribution, age, weight and length with a matching calculated body surface area (calculated by the Haycock formula [15]).

**Table 1:** Anthropometric characteristics and exercise tolerance parameters for rToF patients compared to healthy controls.

	rToF	Controls	p-value*
N	45	45	
Male/female	31/14	31/14	
Age (yrs)	13.9 ± 2.9	13.9 ± 2.8	0.977
W (kg)	50.6 ± 18,3	4.,8 ± 16.3	0.807
L (cm)	157.2 ± 15.0	159.6 ± 18.0	0.364
BSA (m^2^)	1.5 ± 0.3	1.5 ± 0,3	0.990
PVR (yes/no)	20/24		
PS (yes/no)	35/9		
PS gradient (mmHg)	20.4 ±- 8.7		
PI grade:			
0	6		
I	7		
II	10		
III	6		
IV	15		
RVdil (yes/no)	33/6		
FS (%)	35.0 ± 3.7		
TAPSE (mm)	18.1 ± 2.9		
zTAPSE	-2.4 ± 1.8		
QRS-duration (ms)	134.8 ± 19.3		
HRrest (bpm)	89.5 ± 14.5	95.5 ± 12.8	0.041
HRmax (bpm)	174.0 ± 13.8	191.8 ± 9.4	p<0.001
HRres (bpm)	84.5 ± 19.8	96.4 ± 13.1	0.001
RER peak exercise	1.13 ± 0.09	1.14 ± 0.12	0.900
Time (min)	9.23 ± 2.08	12.08 ± 3.03	p<0.001
VO_2_rest (ml/min)	372.3 ± 159.2	349.4 ± 122.4	0.672
VO_2_peak (ml/min)	1658.5 ± 525.3	2110.4 ± 770.8	0.002
VO_2_peak/kg (ml/min/kg)	34.46 ± 8.14	42.77 ± 8.14	p<0.001
PredVO_2_ (%)	81.0 ± 19.7	99.7 ± 13.4	p<0.001
Load (Watt)	112.2 ± 42.4	149.9 ± 65.7	0.002
PredLoad (%)	72.6 ± 27.2	90.9 ± 16.4	p<0.001
VE/VCO_2_-slope	27.37 ± 3.88	25.09 ± 2.88	0.006

*: statistically significant difference p<0.05; Values are expressed as mean ± SD.

*W* Weight, *L* Length, *BSA* Body surface area, *PVR* pulmonary valve replacement, *PS* pulmonary valve stenosis; *PI* pulmonary insufficiency; *RVdil* right ventricular dilatation, *FS* fractional shortening, *TAPSE* tricuspid annular plane systolic excursion, *zTAPSE* calculated zscore of TAPSE, *HRrest* heart rate at rest, *HRmax* maximum heart rate, *HRres* heart rate reserve ie. HRmax-HRrest, *RER* respiratory exchange ratio, *VO*_*2*_*rest* oxygen consumption at rest, *VO*_*2*_*peak* oxygen consumption at peak exercise, *PredVO*_*2*_ VO_2_ expressed in percentage relative to the predicted value, Load maximal obtained workload, *PredLoad* Load expressed in percentage relative to the predicted value, *VE/VCO*_*2*_*-slope* calculate slope of relation between ventilation and carbon dioxide production.

In the rToF group, 20 patients had received a pulmonary valve replacement (PVR) over time. Echocardiography data at the time of the exercise test show that only 6 of them showed no right ventricular dilatation (RVdil). A strong correlation was observed between FS and zTAPSE (r = 0.503; p = 0.001).

### Cardiopulmonary exercise testing

In Table 1 the parameters quantifying exercise tolerance, obtained from the maximal CPET, are presented. Exercise capacity is decreased in patients with rToF; maximal oxygen consumption expressed in absolute values (VO_2_peak) and relative to body weight (VO_2_peak/kg), %predicted VO_2_ (PredVO_2_), maximal load (Load) and %predicted load (PredLoad) are lower compared to the control group. Heart rate at rest (HRrest), maximal heart rate (HRpeak) and heart rate reserve (HRres) were also decreased in the patient group. The VE/VCO2 slope was increased, indicating a lower ventilatory efficiency in rToF patients.

Patients with a better right ventricular function had a higher peak aerobic capacity, as demonstrated by a weak positive correlation between zTAPSE and VO_2_peak and PredVO_2_ (r = 0.338; p = 0.025 and r = 0.371; p = 0.013). No correlation was found between FS and maximal exercise performance parameters.

QRS duration showed a weak negative correlation with PredVO_2_ and PredLoad (r = -0.417; p = 0.005 and r = -0.389; p = 0.010), indicating that a shorter QRS duration could be beneficiary for a better exercise performance [16].

### Recovery kinetics of VO_2_ and VCO_2_

**Fig 2:**
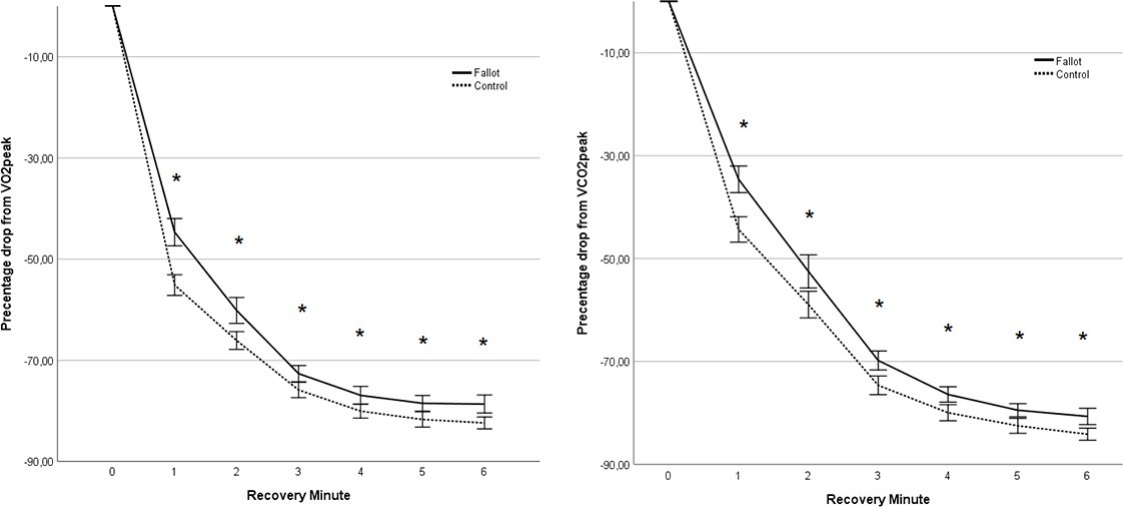
Recovery of VO_2_ (A) and VCO_2_ (B) as percentage of peak value during the 6 min of recovery of the rToFpatients (Fallot) and the control group (Control). *: statistically significant difference p<0.05.

Analyzing the relative changes from peak value per minute, significant main effects of time (p<0.001) and group were found (p<0.001). There was also a significant interaction between group and time (p<0.001). Patients with rToF showed a lower percentage drop from peak value in VO_2_ and VCO_2_ compared to the control group. Post hoc analysis revealed a significant difference at each minute of recovery for both parameters (Fig 2A and 2B, Table 2).

**Table 2:** Recovery parameters of VO_2_ and VCO_2_.

	rToF	Control	p-value*
**Half life time**			
T_1/2_VO_2_ (s)	52.5 ±11.3	44.3 ± 10.5	0.001
T_1/2_VCO (s)_2_	68.3 ± 13.8	59.4 ± 12.1	0.002
**Recovery of VO**_**2**_ **- % drop from peak VO**_**2**_ **per minute**			
	rToF	Control	p-value*
min 1	-44.68 +/- 8.98	-55.13 +/- 6.81	0.000
min 2	-60.14 +/- 8.50	-66.08 +/- 5.82	0.001
min 3	-72.62 +/- 5.33	-75.86 +/- 5.06	0.024
min 4	-76.92 +/- 5.91	-80.04 +/- 4.64	0.040
min 5	-78.49 +/- 5.09	-81.69 +/- 4.85	0.023
min 6	-78.64 +/- 5.79	-82.37 +/- 3.72	0.005
**Recovery of VCO**_**2**_ **- % drop from peak VCO**_**2**_ **per minute**			
	rToF	Control	p-value*
min 1	-34.60 +/- 8.60	-44.34 +/- 8.19	0.000
min 2	-52.50 +/- 10.71	-58.94 +/- 8.63	0.014
min 3	-69.81 +/- 6.15	-74.64 +/- 6.03	0.002
min 4	-76.42 +/- 5.00	-79.96 +/- 5.21	0.009
min 5	-79.49 +/- 4.21	-82.51 +/- 4.68	0.014
min 6	-80.68 +/- 5.14	-84.14 +/- 3.73	0.004

*: statistically significant difference p<0.05; Values are expressed as mean ± SD.

This prolonged recovery of VO2 and VCO2 in children with rToF is confirmed in the higher values of half-life time T_1/2_VO_2_ and T_1/2_VCO_2_, as shown in Table 2.

In the rToF patients, T_1/2_VO_2_ shows a weak negative correlation with VO_2_peak/kg and PredVO_2_ and T_1/2_VCO_2_ with VO_2_peak/kg. Accordingly a weak negative correlation is found between the percentage drop after one minute recovery, Rec1-VO_2_ and Rec1-VCO_2_, and HRmax, VO_2_peak/kg and PredVO_2_ (Table 3).

**Table 3:** Correlations between recovery parameters and exercise tolerance parameters, ventricular function and QRS-duration in rToF patients.

		T_1/2_VO_2_	Rec1-VO2	T_1/2_VCO_2_	Rec1-VCO2
HRmax	R	-0.075	-0.316*	-0.044	-0.349*
	p-value*	0.624	0.035	0.772	0.019
VO2peak/kg	R	-0.307*	-0.484*	-0.298*	-0.481*
	p-value*	0.040	0.001	0.047	0.001
PredictVO2	R	-0.306*	-0.432*	-0.280	-0.439*
	p-value*	0.041	0.003	0.062	0.003
zTAPSE	R	-0.505*	-0.566*	-0.466*	-0.507*
	p-value*	0.000	0.000	0.001	0.000
FS	R	-0.517*	-0.636*	-0.270	-0.373*
	p-value*	0.000	0.000	0.073	0.012
QRS-duration	R	0.188	0.022	0.235	0.094
	p-value*	0.228	0.888	0.130	0.549

*: statistically significant difference p<0.05; r: correlation coefficient.

Moderate negative correlations are found between zTAPSE and T_1/2_VO_2_, Rec1-VO_2_, T_1/2_VCO_2_ and Rec1-VCO_2_, suggesting a higher zTAPSE is related to a faster recovery kinetics of both VO_2_ and VCO_2_. A modest negative correlation is also found between FS and T_1/2_VO_2_ and Rec1-VO_2_, FS correlates weakly with Rec1-VCO_2_.

No correlation can be found between QRS duration and gas exchange recovery kinetics after maximal exercise.

### Recovery kinetics of HR

**Fig 3:**
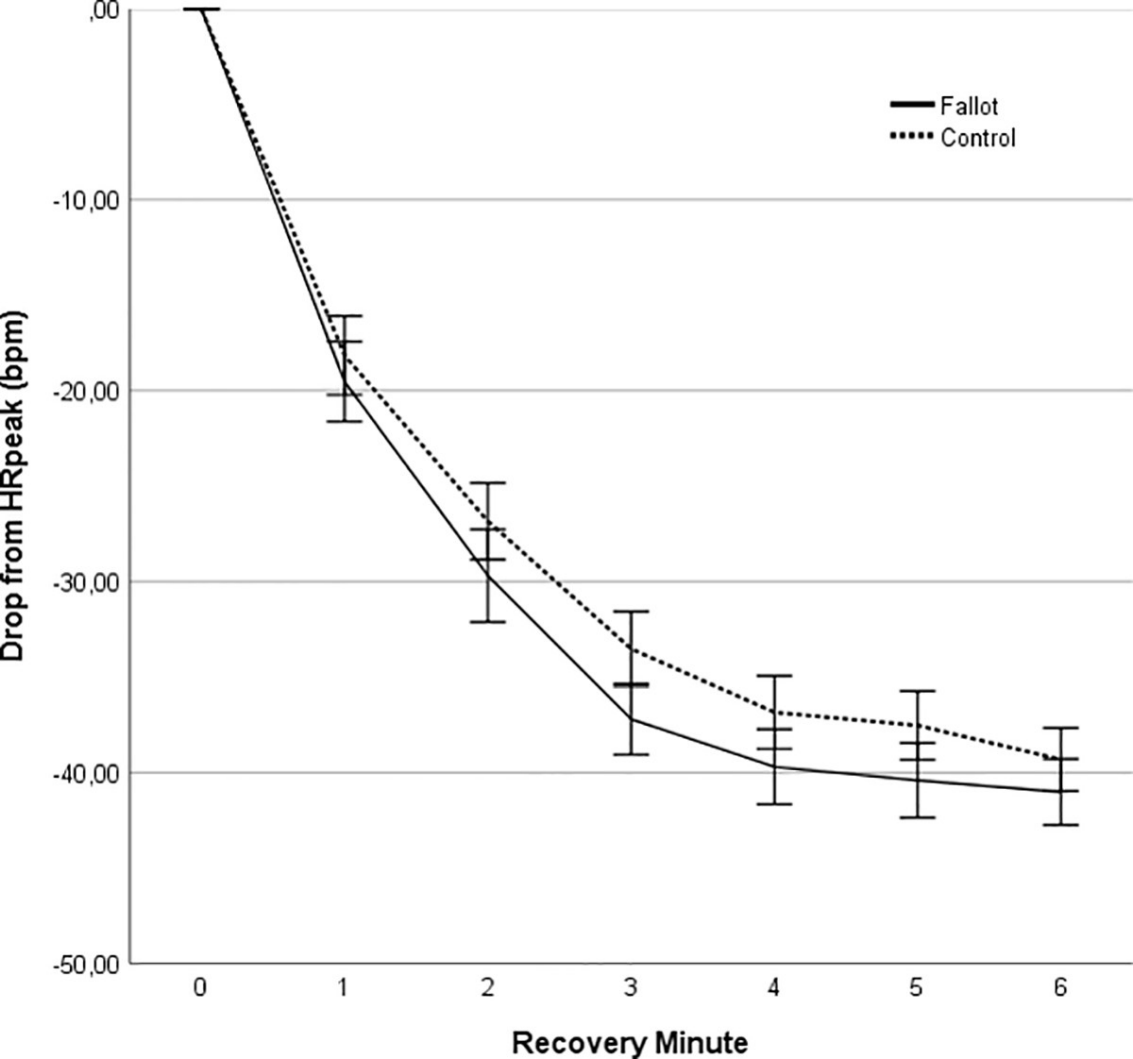
Heart rate recovery (beats per minute) during the 6 min of recovery.

The linear mixed model found a significant main effect of time (p<0.001) but not of group (p = 0.635). There was a significant interaction between group and time (p = 0.037) but post-hoc tests could not show a significant difference in absolute drop of heart rate from peak value in any of the time points in the recovery phase (Table 4, Fig 3).

**Table 4:** Recovery parameters of HR.

	rToF	Control	p-value*
**Half life time**			
T_1/2_HR (s)	61.5 ± 17.7	69.2 ± 28.7	0.130
**Recovery of HR—drop in bpm from peak HR**			
	rToF	Control	p-value*
min 1	-33.93 +/- 12.16	-34.79 +/- 13.35	0.752
min 2	-51.53 +/- 14.06	-51.41 +/- 13.06	0.967
min 3	-64.72 +/- 11.85	-64.17 +/- 12.71	0.833
min 4	-69.15 +/- 12.87	-70.52 +/- 12.35	0.607
min 5	-70.67 +/- 12.58	-72.13 +/- 10.83	0.570
min 6	-71.80 +/- 12.04	-75.64 +/- 9.53	0.115

*: statistically significant difference p<0.05; Values are expressed as mean ± SD.

No difference was found in the half-life time T_1/2_HR between the rToF and the control group (Table 4).

Within the rToF group, no correlations are found between HR recovery kinetics and the parameters describing maximal exercise performance. A weak negative correlation was found between zTAPSE and T_1/2_HR (r = -0.394; p = 0.008).

## Discussion

The main findings of this study demonstrate lower maximal exercise performance and slower recovery of gas exchange parameters after maximal exercise in children with rToF, compared to healthy peers. The two different methods for evaluating recovery, namely percentage of drop after maximal exercise and half-life time calculation of maximal exercise parameters, both demonstrated a delayed kinetics of VO_2_ and VCO_2_. This prolonged recovery is related to diminished exercise performance in this patient group and correlates with zTAPSE and left ventricular function. QRS-duration on the contrary only correlated with maximal exercise performance, not with recovery kinetics.

The evaluation of VO_2_peak during CPET has become an important part in the follow-up after ToF repair. Children and adults after ToF repair have lower maximal oxygen consumption and maximal heart rate [3, 17, 18]. We also found, in line with these other publications, lower VO_2_peak and HRpeak in our patient group. Chronotropic incompetence has proven to be a factor in the exercise limitation in Fallot patients. In the rToF group there was a correlation between HRpeak and VO_2_peak (r = 0.418; p<0.01), confirming the importance of chronotropic incompetence in the maximal exercise performance. Besides chronotropic incompetence, noninvasive cardiac output measurement during exercise also demonstrated lower stroke volume as an important factor of diminished exercise tolerance in rToF [3, 19]. As in other studies [4], we also found a decreased maximal load, test duration and an increased VE/VCO_2_-slope, in the rToF group in comparison with the healthy peers.

The focus of this study is the recovery after maximal exercise performance. Two different methods were used to evaluate recovery kinetics. The determination of half-life parameters and of %drop every minute have both been used to determine the recovery after maximal exercise in literature [5, 8, 14]. Although both methods appeared valuable, it is not investigated whether one of the techniques is superior to the other. This study demonstrates that the two methods used are equally efficient, as can be deducted from a strong correlation between both methods. Furthermore, T1/2VO2 and Rec1-VO2 correlate with VO2peak. This demonstrates both methods are interchangeable. The calculation of the percentage drop at each minute during the recovery generates multiple values to interpret. The half-life calculation provides a single value to describe the entire recovery phase but probably is a technically more challenging method to determine. As in Greutmann et al. [8], we therefore also suggest using the Rec1 as a strong parameter describing the recovery kinetics in this patient population.

In an adult population with heart failure, VO_2_ recovery delay has proven to be an important parameter signalling impaired cardiac output and predicting transplant-free survival [20]. Greutmann et al. [8] demonstrated in 2014 a slower recovery of oxygen consumption (VO_2_) and carbon dioxide production (VCO_2_) in adults with different types of congenital heart disease. The patient group in this study had different types of CHD: transposition, tetralogy of fallot and univentricular heart. No difference could be demonstrated depending on the underlying lesion. Another study evaluated recovery after right ventricular outflow tract reconstruction (RVOTR) in adolescents and young adults in comparison to patients undergoing VSD closure and healthy subjects. This study suggested that patients after RVOTR had delayed post-exercise cardiovascular recovery as a result of right-sided impaired hemodynamics and impaired central autonomous nervous activity [21]. Giardini et al. [9] demonstrated the importance of right ventricular dysfunction and pulmonary regurgitation in young adults and adolescents after ToF repair. They suggested the delayed recovery and diminished exercise performance at this age could be useful to guide pulmonary valve replacement. A delayed recovery pattern after maximal exercise might be an important index of physical fitness and might be important to guide further treatment, as shown by Lurz et al. [10] in an adolescent population of a mixed group of cardiopathies necessitating pulmonary valvar replacement.

We compared the recovery of VO_2_ and VCO_2_ in young rToF patients and found significantly slower recovery using both methods in comparison to children without cardiac defects. T_1/2_VO_2_, T_1/2_VCO_2_ and the %drop of VO_2_ and VCO_2_ after each minute of recovery demonstrate significantly different patterns of recovery in comparison to healthy children. The VO_2_ decline after recovery is dependent on the magnitude of oxygen debt and the rate of oxygen delivery as determined by cardiac output during recovery. Oxygen debt is defined [6, 22] by the deviation from homeostasis, i.e. the amount of lactate accumulation. Since we found no difference in RER at peak exercise between rToF children and healthy controls, we can presume they reached the same level of physical exhaustion. Therefore the strong correlation between VO2peak and recovery indicates cardiac output probably is the more determining factor in the recovery kinetics. This was also assumed by Singh et al. [7] who found similar results in a study comparing different groups of children with CHD with healthy controls.

Studies in several adult populations describe the importance of heart rate recovery (HRR) as a tool to assess cardiac autonomic nervous activity and emphasize its possible prognostic value [23–26]. In contradiction to Greutmann et al, who describe a prolonged heart rate recovery in young adults with CHD compared to healthy controls, we did not find a delay in heart rate recovery kinetics in the rToF patients. However, this might be due to the fact that our population is younger. It is known that children recover faster from exercise than adults [6] and have more HR variability [27].

Singh et al. [27] demonstrated that HR recovery, defined as 1min drop from HRmax, prolongs with age in children and children with lower exercise endurance have a slower HRR. This could not be confirmed in our rToF group, where no correlations were found between the parameters describing heart rate recovery and VO_2_peak/kg, PredVO_2_ and PredLoad.

To better understand the difference between patient and control group in gas exchange recovery delay after maximal exercise, we analysed correlations between recovery parameters and left ventricular systolic function, zTAPSE, QRS duration and RV dilatation. In our study, QRS-duration correlates strongly with VO_2_peak, both parameters have proven to be strong predictors of event-free survival in rToF [4]. However, no correlation could be found between QRS-duration and VO_2_ recovery kinetics. These findings indicate that QRS duration, and hence the RV/LV synchronicity, seems to be an important factor contributing to the ability to perform the maximal step in exercise performance, but has less influence in the following recovery. Budts et al. [16] also demonstrated that adult rToF-patients with a shorter QRS duration have higher maximal exercise performance. On the other hand, a significant correlation was found between zTAPSE and LV fractional shortening and T_1/2_VO_2_ and Rec1-VO_2_. This demonstrates the importance of good left ventricular function and right ventricular wall motion in the recovery process after exercise. As we did not withhold differences in HR recovery between both groups, LV and RV function as the more important factors influencing the cardiac output seem to play a determining role in the repayment of oxygen debt after a maximal exercise. Given these findings thorough assessment of the recovery phase might contribute to a better decision making when evaluating rToF patients for further treatment options.

### Study limitations

This study has some limitations. This is a retrospective monocentric study; in the future, larger multicentric studies would be necessary to determine the value of VO_2_-recovery as possible prognostic parameter. The evaluation of the recovery kinetics after exercise can be time-consuming; however, we propose to use Rec1 as the easiest definable parameter for follow-up. Also, as this is a retrospective study, we did not have cardiac MRI investigations of the patient group, which would give a more reliable estimation of RV function and dilatation.

## Conclusion

Children after ToF repair have diminished exercise performance and slower recovery of VO_2_ and VCO_2_ compared to healthy peers. Children with more diminished exercise capacity within this patient group have the slowest recovery patterns of these parameters. Whereas QRS-duration is correlated with maximal oxygen consumption, left ventricular function and TAPSE are interrelated with recovery and oxygen debt repayment after maximal exercise. These findings demonstrate the importance of LV and RV function in the recovery process of physical activities.

## Supporting information

S1 Dataset (XLSX)
